# Special Section Guest Editorial: Thirty Years of Multiphoton Microscopy in the Biomedical Sciences

**DOI:** 10.1117/1.JBO.25.1.014501

**Published:** 2020-01-31

**Authors:** Ammasi Periasamy, Karsten König, Peter So

**Affiliations:** a University of Virginia, W.M. Keck Center for Cellular Imaging, Departments of Biology and Biomedical Engineering, Charlottesville, Virginia, United States; b Saarland University, Department of Biophotonics and Laser Technologies, Saarbrücken, Germany; c JenLab GmbH, Berlin, Germany; d Massachusetts Institute of Technology, Department of Biological Engineering, Cambridge, Massachusetts, United States

## Abstract

JBO guest editors introduce the Special Section Celebrating Thirty Years of Multiphoton Microscopy in the Biomedical Sciences.

After the invention of the microscope by Antoni van Leeuwenhoek in the 1670s, the ability to investigate structures and mechanisms at the microscopic level has allowed scientists to better grasp the often misunderstood relationship between microscopic and macroscopic behavior. Furthermore, imaging specimens through a microscope preserves the temporal and spatial relationships that are frequently lost in traditional biochemical techniques, and produces a two- or three- or four-dimensional resolution that other laboratory methods cannot. There are several microscope techniques that have been established for cellular imaging; among them, the benefits of fluorescence microscopy (FM) techniques are numerous. FM plays a vital role in the biological and biomedical sciences where fluorescence probe specificity and sensitivity can provide important information regarding the biochemical, biophysical, and structural status of cells.^1^ Recent advancements in light sources, detection systems, data acquisition methods, image analysis, and display methods have further broadened the applications in which fluorescence microscopy can successfully be applied.^2^ In particular, the rapid advancements of confocal and multiphoton microscopy over the past three decades have been major contributors to our understanding of dynamic processes in cells, tissues, and live animals.^3^^–^^5^

Considering the historical perspective of multiphoton microscopy, in 1931, Maria Göppert-Mayer reported the quantum mechanical formulation of two-photon molecular excitation in her doctoral thesis.^6^ Two-photon excited fluorescence was finally demonstrated by Kaiser and Garrett^7^ shortly after the invention of the laser in 1960. Sheppard and co-workers developed the SHG microscope^8^ for solid state specimens and Denk et al. developed a non-linear fluorescence microscope and first showed that it can be applied to image biological system non-invasively.^9^

Laser scanning multiphoton fluorescence microscopy was developed by Denk et al. in 1990.^9^ Since then, the usage and the development of multiphoton microscopy has increased tremendously (see Fig. 1). More importantly, the commercialization of multiphoton imaging systems by Bio-Rad did increase awareness and application of this technology in biomedical imaging. It should also be recognized that laser companies (Spectra Physics and Coherent) played a key role in introducing tunable (700-1100 nm; currently from 680 to 1300 nm) femtosecond infrared-pulsed laser systems (Ti: sapphire) for multiphoton imaging. Moreover, the future will bring even easier-to-use equipment and increased sensitivity, which will allow greater flexibility in the simultaneous imaging of multiple fluorophores while images are collected over time and at greater depths inside tissue/live animals.

**Fig. 1:**
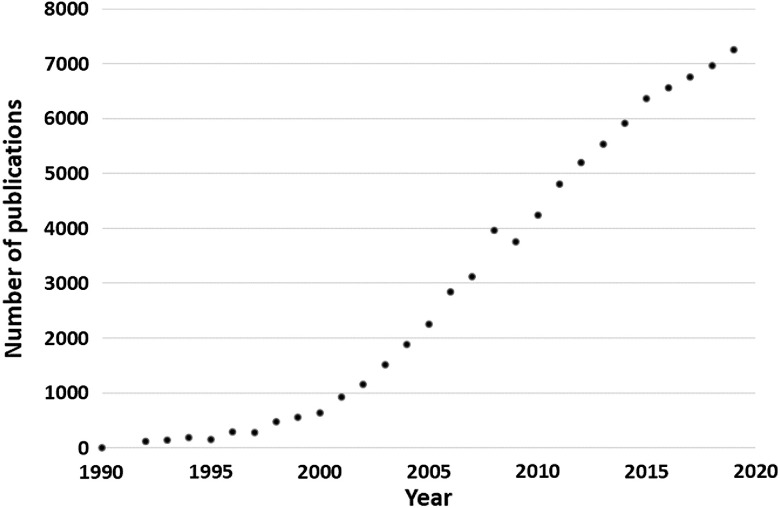
Number of publications in multiphoton microscopy since 1990.

To describe the multiphoton excitation process in brief, an infrared femtosecond pulsed laser is required to create multiphoton absorption in a biological sample. Multiphoton excitation occurs at only a single, diffraction-limited spot where the photon flux is great enough to allow absorption of more than one photon. For example, for one-photon excitation, a wavelength of 480 nm CW (continuous wave) is selected for GFP, but for two-photon excitation, a single 960 nm wavelength from a Ti: sapphire pulsed laser is used. For most of the fluorophores, the two-photon absorption cross-section is blue shifted. Multiphoton images have better signal-to-noise compared to confocal images because of a considerably reduced amount of light scattering due to infrared wavelengths used for 2-photon imaging. In addition, autofluorescence and photobleaching are minimized, as there is no absorption throughout the specimen beyond the focal volume or focal plane.

The contributions and usage of multiphoton microscopy in the areas of biological and biomedical sciences over the last 30 years have generated a high level of interest among scientists and students. As shown in Fig. 1, there has been tremendous increase in publications in the area of technology development and the usage of the multiphoton microscopy in various areas of research. To mention a few, on the technology development side, there are a number of miniaturized and fiber-optics-based two-photon microscopy developments, three-photon microscopy^10^^,^^11^ and longer wavelength applications to image deep inside tissue and animal specimens,^12^ and second and third harmonic generation microscopy in tissue and live animals.^13^ There is also two- and three-photon lifetime imaging, metabolism-related imaging (NADH, FAD, tryptophan);^14^^–^^18^ calcium, pH, and oxygen^19^ membrane potential measurements; amino acid tryptophan imaging; multiphoton tomography; coherent anti-Stokes Raman scattering (CARS) imaging;^20^ and single molecule detection, super resolution.^21^^–^^23^ Closer to human pathologies there is stem cell research, clinical applications in surgery, cancer diagnosis and treatment, and very specifically, skin cancer diagnosis, best highlighted by the commercialization of multiphoton microscopy by JenLab for skin cancer diagnosis.^24^
